# Recent Advances in Carbon and Nitrogen Metabolism in C3 Plants

**DOI:** 10.3390/ijms22010318

**Published:** 2020-12-30

**Authors:** Marouane Baslam, Toshiaki Mitsui, Kuni Sueyoshi, Takuji Ohyama

**Affiliations:** 1 Laboratory of Biochemistry, Faculty of Agriculture, Niigata University, Niigata 950-2181, Japan; mbaslam@gs.niigata-u.ac.jp (M.B.); t.mitsui@agr.niigata-u.ac.jp (T.M.); 2 Department of Life and Food Sciences, Graduate School of Science and Technology, Niigata University, Niigata 950-2181, Japan; sueyoshi@agr.niigata-u.ac.jp; 3 Faculty of Applied Biosciences, Tokyo University of Agriculture, Tokyo 156-8502, Japan

**Keywords:** C and N interactions, fixation, assimilation, transport, plant-microbiome interactions, omics, starch, stress

## Abstract

C and N are the most important essential elements constituting organic compounds in plants. The shoots and roots depend on each other by exchanging C and N through the xylem and phloem transport systems. Complex mechanisms regulate C and N metabolism to optimize plant growth, agricultural crop production, and maintenance of the agroecosystem. In this paper, we cover the recent advances in understanding C and N metabolism, regulation, and transport in plants, as well as their underlying molecular mechanisms. Special emphasis is given to the mechanisms of starch metabolism in plastids and the changes in responses to environmental stress that were previously overlooked, since these changes provide an essential store of C that fuels plant metabolism and growth. We present general insights into the system biology approaches that have expanded our understanding of core biological questions related to C and N metabolism. Finally, this review synthesizes recent advances in our understanding of the trade-off concept that links C and N status to the plant’s response to microorganisms.

## 1. Metabolism and Transport of C and N in C3 Plants

C and N are the most important essential elements in plants, animals, and microorganisms. They act as limiting factors for plant growth and crop yield, which makes their metabolism and transport important for agricultural practices. Plant roots absorb water and nutrients from the soil and transport them to the shoots via xylem vessels in the roots, stems, and leaves. Transpiration through the stomata in the leaves and root pressure are two driving forces the “pull” the water and dissolved nutrients upward from the roots to the leaves against the force of gravity. Plant roots generally absorb N in the form of ammonium and nitrate, the most dominant available N compounds in upland soil except for soil organic matter. Plant roots can absorb some organic N compounds, such as amino acids, although their contribution is generally low under natural conditions. Recent works showed the contribution of amino acid root uptake, such as the uptake of acidic aspartate and glutamate, as well as neutral alanine, glycine, serine, threonine, and isoleucine, to the growth and yield of rice [1,2] and ^15^N-labeled glutamine in soybean plants [3].

**Figure 1:**
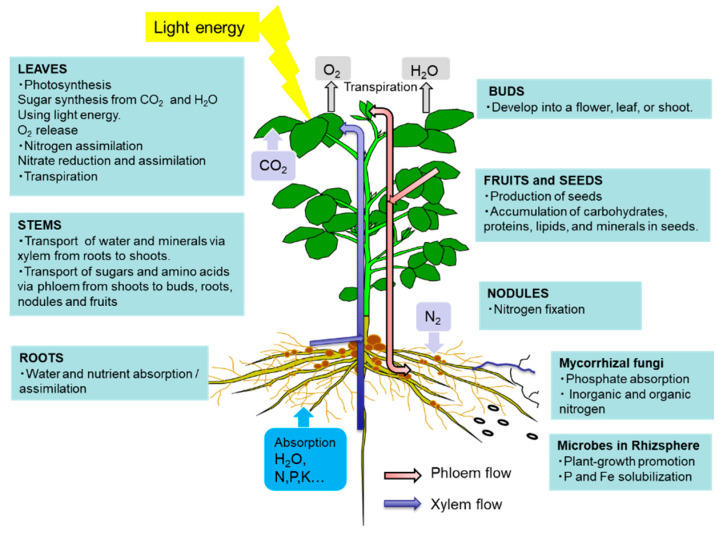
Overview of the C and N flow among plant organs. Plants use the xylem–phloem system to exchange nutrients and the information on the status of water and mineral nutrient acquisition by the root system and the growth requirements of the shoot organs. The C flow from shoots supports roots, symbionts (i.e., fungi and bacteria) that provide plants with nutrients and other benefits, and young (sink) organs, such as buds, flowers, and fruits, via the phloem (noted in red arrow). Xylem (noted in blue arrow) conveys water and nutrient from the roots to the rest of the plant and also provides physical support. Several of the displayed systems may occur independently in nature (e.g., nodules, mycorrhizal fungi, and plant growth-promoting rhizobacteria (PGPR)) and/or may not be present at the same time on the root. Bud, flower, and fruit differentiation and development may appear sequentially on the shoots.

The leaves play a role in photosynthesis, which uses light energy to produce carbohydrates from the atmospheric CO_2_ and root-derived H_2_O while simultaneously releasing O_2_ as a by-product (Figure 1). Sucrose is the main form of photoassimilate transported from the mature leaves to the roots, symbionts, and growing sink organs, such as buds, flowers, and fruits, via the phloem. Leaves play an important role in N metabolic processes, such as nitrate reduction, assimilation, and amino acid transport, to support other organs.

**Figure 2:**
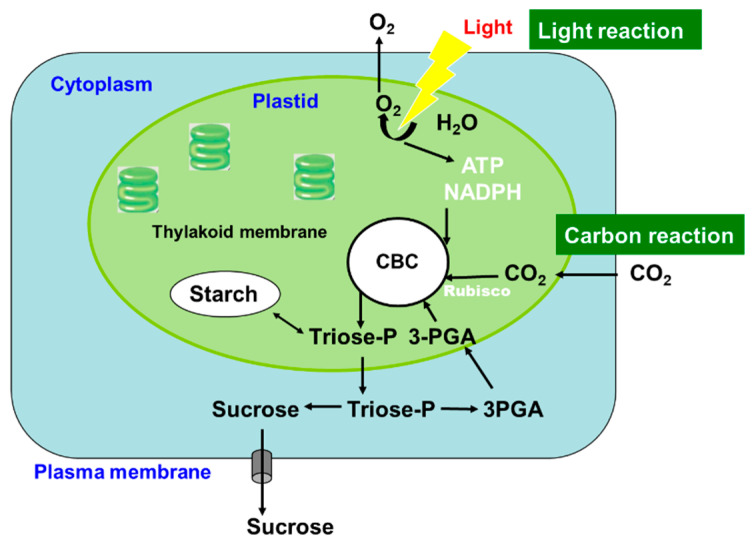
Photosynthesis in C3 plant leaves. 3-PGA: 3-phosphoglycerate and Rubisco: Ribulose-1,5-bisphosphate carboxylase/oxygenase.

**Figure 3:**
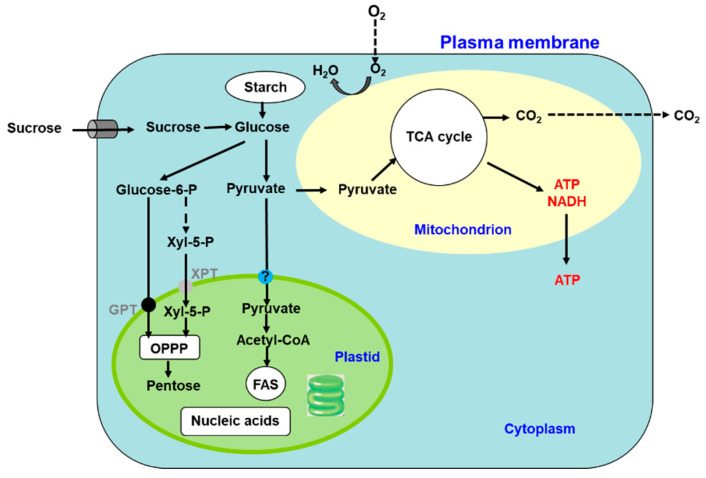
Respiration and C utilization in a plant cell. AA: Amino acid; FAS: Fatty acid synthesis; GPT (Glucose phosphate transporter): OPPP: Oxidative pentose phosphate pathway; TCA: Tricarboxylic acid: XPT: Xylose phosphate transporter; XPT: Xylulose 5-phosphate/phosphate translocator; and Xyl-5-P: Xylulose 5-phosphate.

Photosynthesis is a complex series of reactions that involve light absorption, energy conversion, electron transfer (light reaction), and enzymatic pathways that convert CO_2_ and H_2_O to carbohydrates (carbon reaction) [4]. Figure 2 shows an outline of photosynthesis in C3 plants [5]. In addition to C3 plants, plants with C4 and CAM types of photosynthesis are widely distributed, but we do not describe these types of plants in this review. Chlorophyll molecules absorb light energy in the thylakoid membrane of chloroplasts, where photosystems I and II convert the electron excitation energy into chemical energy (light reaction). Subsequently, the ATP and NADPH produced in the light reaction are used for CO_2_ fixation and assimilation in the Calvin cycle, where the first step is catalysis by the ribulose-1,5-bisphosphate carboxylase/oxidase (Rubisco) enzyme (carbon reaction). Thereafter, the triose-P (dihydroxyacetone-P, glyceraldehyde-P) produced in the chloroplasts is transported to the cytosol and used for sucrose synthesis [6]. Extra triose-P produced during daytime can be stored in the form of starch in chloroplasts, which can then be degraded to provide sugars during the night. Another possibility for storing extra triose-P is to generate chlorogenic acids via the shikimate pathway [7]. Through this metabolic pathway, phenylpropanoid, cellulose, hemicellulose, and lignin biosynthesis can be considered as major end-products of C metabolism. Plant cells use glucose-P derived from sucrose or starch degradation to generate energy and synthesize C materials for organic compounds (Figure 3). Glucose is metabolized to pyruvate through glycolysis in the cell cytoplasm. Pyruvate is then transported to the mitochondria, where it is further metabolized to CO_2_ through the tricarboxylic acid cycle. This process produces the energy compound ATP and the reductive compound NADH, which are used for various cellular processes. Glucose-6-phosphate is transported to the plastids and metabolized through the pentose phosphate pathway, producing pentose and NADPH. Pentoses are used for nucleic acid (DNA and RNA) synthesis, and NADPH is required for lipid synthesis.

**Figure 4:**
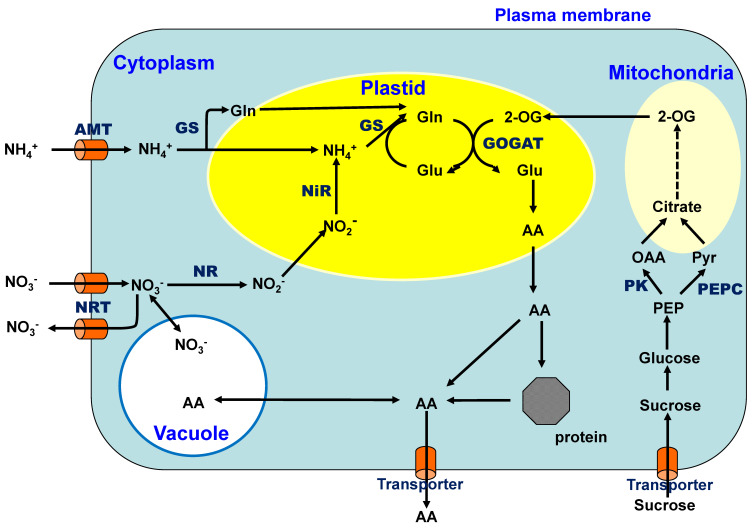
Ammonium and nitrate absorption and assimilation related to carbon metabolism in a plant root cell. NR: Nitrate reductase; NiR: Nitrite reductase; GS: Glutamine synthetase; GOGAT: Glutamate synthase AMT: Ammonium transporter; NRT: Nitrate transporter: PEP: Phosphoenolpyruvate; PEPC: PEP carboxylase; and PK: Pyruvate kinase.

Nitrate is the most abundant inorganic N form in upland fields because ammonium is readily oxidized to nitrate by nitrifying bacteria under aerobic conditions [8]. Nitrate is absorbed through the nitrate transporters located in the plasma membranes of the cells (Figure 4) and is reduced to nitrite by the enzyme nitrate reductase (NR) in the cytoplasm using one mole of NADH or NADPH as a reductant. The nitrite ions are incorporated into the plastids and further reduced to ammonium ions via enzyme nitrite reductase (NiR) using six moles of the reduced form of ferredoxin. The ammonium ion is initially combined with glutamic acid (Glu) and assimilated into glutamine (Gln) by the enzyme glutamine synthetase (GS), which consumes one mole of ATP. The amide group of Gln is then transferred to an organic acid, 2-oxoglutarate (2-OG), by glutamate synthase (GOGAT) in plastids using two moles of reduced ferredoxin [9]. The amino group of Glu is transferred to an organic acid and produces many amino acids (AA) via transaminases. Previously, ammonium was considered to be initially assimilated into Glu by glutamate dehydrogenase (GDH). However, the GS/GOGAT cycle has been confirmed as the principal route of ammonium assimilation in plants from ammonium absorption, nitrate reduction, or N_2_ fixation [9,10].

As nitrate availability in the field varies widely [11], nitrate reductase activity is finely regulated by various internal compounds and environmental conditions at the transcription, translation, and protein degradation levels [9]. Nitrates and metabolites such as Gln, sucrose, cytokinins, and light have been shown to be regulatory factors for NR [9,12] Nitrate reduction mainly occurs under light conditions during daytime due to the beneficial use of reductants produced by photosynthesis for energy conservation. The NR mRNA concentration cycle in a diurnal rhythm is driven by the circadian clocks. Lillo et al. (2004) suggested that the endogenous formation of reduced N compounds is involved in the diurnal rhythmicity of the NR mRNA level [13]. The rapid and reversible regulation of NR by inactivation/activation occurs when plants are exposed to light/dark transition conditions. After transferring plants from light to dark, NR is phosphorylated by calcium-dependent protein kinases and inactivated by the subsequent binding of 14-3-3 proteins. When plants are re-exposed to light, NR is dephosphorylated by phosphatases and recovers its activity via release from the 14-3-3 protein [9].

Amides and amino acids, mainly asparagine (Asn) and Gln, are used for N transport through the xylem and phloem in many plants. Gln and Asn are suitable for N transport and storage due to the presence of two N atoms in one molecule and their higher solubility among the amino acids [10]. Moreover, arginine—which has the highest N/C ratio—especially suitable as an N storage form of organic nitrogen [14]. Notably, some legumes use ureides instead of Asn or Gln to transport the bulk of their fixed N from nodules.

**Figure 5:**
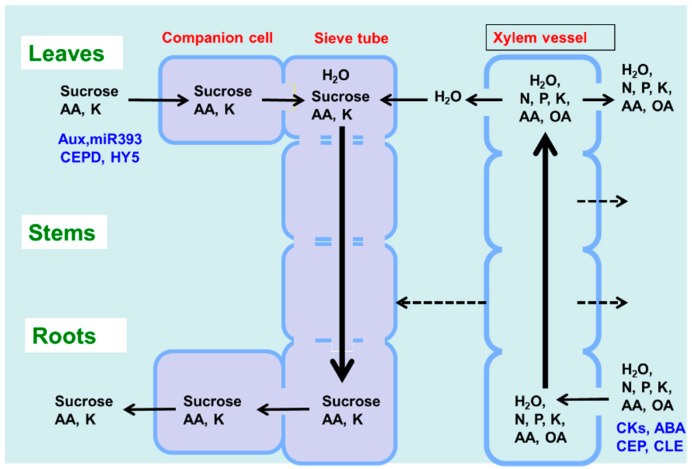
Transport of C and N and regulatory signals through the phloem and xylem. OA: Organic acid; CKs: Cytokinins; ABA: Abscisic acid; CEP: C-terminally encoded peptide; CEPD: CEP downstream; HY5: Elongated hypocotyl 5; and miR393: MicroRNA393.

As shown in Figure 1, there are two routes by which materials are transported among plant organs in the vascular system of the xylem and phloem. Figure 5 illustrates the model of xylem and phloem transport in the roots, stems, and leaves. The xylem vessels are a system of pipes made up of dead cells, through which water and absorbed nutrients, such as N, P, K, Ca, Mg, and minor elements, are transported from the roots to the shoots. The major driving forces for the upward movement against gravity are transpiration and root pressure. During the daytime, the stomata on the leaf surface are opened to allow the entry of CO_2_ from the air. At the same time, the water inside the leaf is evaporated through the open stomata to the air. As a result, the water potential in the leaves decreases, and the difference in the water potential of the roots and leaves causes an upward movement of the fluid in the xylem vessels. The transpiration from leaves creates negative pressure in the xylem, and the stem becomes slightly thinner during the day compared to the night. Another driving force for the upward movement of water is the root pressure. After cutting the shoots, the cut stem bleeds a solution, which is called xylem sap, via the root pressure that results from the gradient in hydrostatic pressure from the soil solution to the root cells [15].

Photoassimilates (mainly sucrose), amino acids, and minerals such as K are transported bi-directionally from the leaves to the roots, buds, and fruits via a sieve tube in the phloem. This sieve tube is a channel of sieve-tube cells connected end-to-end, similar to xylem vessels. These sieve-tube cells feature cytoplasm surrounded by the plasma membrane, although the nucleus is lost, and new protein synthesis does not occur. In a previous study, the movement of radioisotopes from the leaves to the roots showed that the maximum velocity in the phloem is 22 cm/h and that in the xylem is 147 cm/h in the fruit stalks of white lupin [16]. The motivating force of phloem transport was studied using the pressure-flow theory proposed by Ernst Munch in 1930 [17]. Sucrose is loaded in the sieve tube through companion cells, and the concentration of sucrose in the phloem sap becomes extraordinarily high at ca. 10–30% (*w*/*v*) [17]. This high concentration of sucrose in the source organ causes high osmotic pressure, and the water in the leaf apoplast then flows into the sieve tube. On the other hand, sucrose is also unloaded into the sink organs, such as roots, causing a decrease in sucrose concentration and osmotic pressure. The gradient of the osmotic pressure between the source and sink organs is considered to be a driving force for the constant flow of phloem sap. However, the cooling of parts of the stems of some plant species causes a reversible decrease in phloem transport, suggesting that some biological processes, such as cytoplasmic streaming, may influence phloem transport.

The xylem sap contains relatively higher concentrations of amino acids (0.1–2 g/L), mostly Asn, Gln, aspartic acid (Asp), and Glu, as well as inorganic ions (0.2–4 g/L), such as K, P, N, Ca, Mg, and minor elements [17]. When nitrate is applied to the plants, it can become a significant component of the xylem sap, although there is considerable variation in the sites of nitrate reduction between the roots and shoots among plant species. Generally, sucrose is absent in the xylem sap. However, some organic acids, such as malate, are present in relatively abundant amounts and may play a role in maintaining the cation–anion balance. The composition of the phloem sap is quite different from that of the xylem sap, with concentrations of sucrose, amino acids, and K being remarkably higher in the former compared to the latter. The xylem vessels are not entirely closed and leak water and solutes into the stems during transport (dotted line in Figure 5). In the transport of amino acids from the roots to the shoots in soybean, approximately 21–33% of the transport occurs in the stems, and approximately 60–73% occurs in the leaf blades [18]. In vascular bundles, the phloem and xylem are separated by only a few cells [15]. In the stem nodes, intensive xylem-to-phloem transfer occurs, especially in the nodes between the roots and shoots, where the two vascular bundles are complexly connected [19].

The xylem and phloem transport not only nutrients but also various signal compounds, some of which are shown in Figure 5 (in blue) [20]. Phytohormones, cytokinins, abscisic acid (ABA), and C-terminally encoded peptide (CEP) and CLAVATA3-like (CLE) peptides are transported through the xylem vessels, whereas auxin, microRNA, CEP downstream (CEPD), and elongated hypocotyl 5 (HY5) proteins are transported from the shoots to the roots via the phloem.

## 2. Regulation and Interaction of C and N Metabolism

The exchange of C and N between the shoots and roots through the xylem and phloem is crucial. N plays a significant role in C metabolism due to its function in protein synthesis. Likewise, C compounds are essential for N absorption, nitrate reduction, N_2_ fixation, and amino acid metabolism to generate C skeletons, metabolic energy, and reductants. Since a significant amount of fixed C is required to provide the C skeletons that act as acceptors for assimilating N into amino acids to form proteins and other nitrogenous compounds, a correlation between carbohydrate content increases and the downregulation of genes involved in photosynthesis and N metabolism has been reported [21]. N is a limiting factor for the growth and yield of most of crops [22]. When N availability is low, plant growth is stunted, and the leaves show chlorosis because of a decrease in the photosynthetic pigment chlorophyll. Besides under N deficiency conditions, the N in mature leaves is also mobilized to the growing parts and enhances the senescence of older leaves [15]. An increase in the N supply stimulates plant growth, making the plant taller, and also delays senescence. Furthermore, the N supply changes the plant morphology, as shown by an increase in the shoot to root dry weight ratio of both annual and perennial plant species [15]. However, excess N in the soil can be harmful to plants because it stimulates the overgrowth of vegetative organs and inhibits the growth of reproductive organs, ultimately decreasing crop yields [23,24]. The root architecture is modified by the levels and the placement of N in the soil. Yashima et al. [25] showed that soybean plants cultivated under an N-free nutrient solution have longer roots than plants with a nitrate supply.

The green revolution drastically increased cereal crop yields by up to two to four times by selecting semi-dwarf varieties, tolerant to the application of high amounts of chemical N fertilizers over original domestic varieties, ultimately showing overgrowth and lodging under high-N conditions. Li et al. [26] reported that increasing the productivity of the green revolution varieties of cereals increased environmental damage because of the increased application of N fertilizers and that it is vital to improve N-use efficiency (NUE) to solve environmental pollution problems. The authors showed that the antagonistic activities and physical interactions of the rice growth-regulating factor4 (GRF4) transcription factor (TF) and the growth repressing DELLA (Aspartic acid-glutamic acid-leucine-leucine-alanine) protein are required for the homeostatic regulation of C and N metabolism and growth. In WT wheat, bioactive gibberellins (GAs) promote plant growth by stimulating the degradation of DELLA proteins. The semi-dwarf green revolution varieties of cereals (GRVs), conferred by DELLA accumulation, are resistant to GA-stimulated DELLA destruction, whereas the rice GRV mutant reduces the abundance of bioactive GAs [26].

In addition to playing a role in nutrition, C and N may act as signals to regulate nutrient absorption, assimilation, photosynthesis, and eventually plant growth and crop yield through the expression of related genes, enzymatic activities, and signal transduction networks [27]. Nitrate is absorbed by nitrate transporters (NRTs), and ammonium by ammonium transporters (AMTs), in the plasma membrane. The nitrate transporter, nitrate reductase, and glutamine synthetase genes were observed to be induced in sugar-depleted Arabidopsis supplied with extra exogenous sugar [28]. Sugar also induces the expression of NRT genes in Arabidopsis [29]. The application of nitrate to N-depleted plants leads to the direct induction of the glycolytic pathway genes that are involved in the synthesis of the C-skeleton for amino acid production during nitrate assimilation [30,31,32]. Moreover, a reduction in sugar level leads to the inhibition of nitrate assimilation in tobacco plants [33].

Three genes, nitrate transporters (LIN1/NRT2.1), glutamate receptor (GLR1.1), and a methyltransferase named oversensitive to sugar 1 (OSU1), were found to be involved in C–N signaling in Arabidopsis [34]. Zhang et al. [35] reported the C/N metabolic balance in cyanobacteria as a simple model of photosynthetic organisms. It was found that 2-phosphoglycolate (2-PG) derived from the oxygenase activity of Rubisco and 2-OG from the tricarboxylic acid (TCA) cycle act as C and N starvation signals, respectively. The levels of 2-PG and 2-OG are inversely correlated, and their ratio reflects the C/N metabolic balance. Under N limitations or C oversupply, the level of 2-OG increases, whereas that of 2-PG decreases. The 2-OG/dimeric global TF (NtcA) complex activates the genes involved in N uptake and assimilation, while the 2-OG/NdhR (Rubisco operon transcriptional regulator) complex represses the genes related to the CO_2_-concentrating mechanism, decreasing C uptake. The authors noted that the signaling role of 2-OG and 2-PG in C/N balance is likely conserved in other photosynthetic organisms. The role of 2-OG as a master regulator metabolite for regulating C/N metabolic balance has also been reported in *Escherichia coli* [36].

Glutamate plays an essential role in the excitation of neurotransmitters in the mammalian nervous system via a family of ionotropic glutamate receptors (iGluRs) [37]. In 1998, it was found that plants contain a family of glutamate receptor-like (GLR) genes that are related to the mammalian iGluRs [38]. In animals, iGluRs operate as Glu- and Gly-gated non-selective cation channels allowing the uptake of K^+^, Na^+^, and Ca^2+^ into neurons, but plant GLR receptors have much broader amino acid specificity via Ala, Asn, Cys, Glu, Gly, and Ser [31]. Gent and Forde [34] noted that potential N sensory systems include the target of the rapamycin (TOR) signaling pathway, the general control non-derepressible 2 (GCN2) pathway, the plastidic PII-dependent pathway, and the family of glutamate-like receptors (GLRs). However, despite significant recent progress in elucidating the functions and modes of action of these signaling systems, there is still much uncertainty about the extent to which they contribute to the process by which plants monitor their N status.

Nutrient availability controls plant growth and development, which are finely tuned by hormonal signals [39]. The nitrate transporter NRT1.1 was found to be an auxin-responsive gene, and the promoter activity of *NRT1.1* was reported to be induced by auxin. Auxin signal transduction is mediated by the auxin receptor TIR1 (Transport inhibitor response 1); further, its homologous gene AFB3 (Auxin signalling F-Box 3), two auxin carriers (At2g17500 and At1g76520), and four efflux auxin transporters (PIN1, PIN2, PIN4, and PIN7) have been shown to be controlled at the transcriptional level by C and/or N treatments. The nitrate responsive microRNA393 (miR393): AFB3 module can integrate external nitrate availability and the internal N status in plants [40]. The roles of non-coding RNAs in response to N availability in plants have also been reviewed recently [41]. In addition, NRT1.1 has been shown to be involved in nitrate-repressed auxin transport [42]. An increase in nitrate supply increases the nitrate and cytokinin concentrations in the xylem exudate [43]. Sakakibara et al. reviewed the interaction between N and cytokinin in the regulation of metabolism and development [44]. ABA signaling is also involved in the integration of C/N nutrient and environmental signals [45]. Recently, Chen et al. [46] reported that Arabidopsis HY5 is a shoot-to-root mobile signal that mediates the light promotion of root growth and nitrate uptake. The shoot-derived HY5 auto-activates root HY5 and promotes root nitrate uptake by activating NRT2.1. In the shoots, HY5 promotes C assimilation and translocation, whereas in the root, the HY5 activation of NRT2.1 expression and nitrate uptake are potentiated by increased sucrose levels.

## 3. C and N Metabolism in the Presence of Plant-Microorganism Interactions

Plants release substantial amounts of complex photosynthetically derived C (20% to 50%) as exfoliates and root exudates (e.g., organic acids, flavonoids) into the rhizosphere [47], an input that plays a crucial factor in the increasing microbial abundance and activity in the rhizosphere compared to bulk soil. The extent to which this C flow (together with N assimilation and partitioning) is integrated into root and rhizosphere functions is of great interest for both basic (model plants and ecology) and applied sciences to increase crop yield and engage in plant disease control and/or bioremediation, thereby meeting our dramatically increasing demand for food. This section does not intend to describe the details of exudates (i.e., their composition and mechanisms), trait-based microbial strategies, or soil C and N cycles. For these topics, the reader is directed to the reports in [48,49,50,51,52,53,54,55]. Here, we present an overview of the microbial impacts on the organization and functioning of C and N metabolism in plants. According to their lifestyles (their involvement in the plant host), microorganisms can be divided into two groups, detrimental pathogen species (biotrophic to necrotrophic) or, more commonly, those with neutral or mutualistic interactions (e.g., below-ground, such as mycorrhiza, plant growth-promoting rhizobacterias (PGPRs), and rhizobia and above-ground, such as endophytes or epiphytes). In this context, the types of plant–-microbe interactions encompass competition, commensalism, mutualism, and parasitism, which can affect C and N metabolism. Both mutualistic and pathogenic microbes can colonize either sink or source organs and interfere with the source–sink balance due to their required sugar supply from host plants to the heterotrophic colonizing agent.

The beneficial plant–microbe interactions positively affecting plant growth, obtained via PGPR, pseudomonas, bacilli, trichoderma, diazotrophs, arbuscular mycorrhizal fungi (AMF), phosphate-solubilizing fungi, and bacteria or cellulose-degrading bacteria, are dependent on many external factors, including photosynthesis activity, plant size, and soil conditions [56,57]. Mycorrhizae do not have strict host specificity compared to rhizobia and are more widely distributed than root nodules throughout plant–microbe interactions; more than 90% of terrestrial plant species can establish mutualistic symbiotic associations with AMF. C handling is a fundamental aspect of this symbiosis. AMF derive most of their C from the host plant by increasing plant biomass and photosynthesis and directing the flow of a significant fraction of the host plant’s photoassimilates. Between 20% to 40% of the photoassimilates in host plants flow to mycorrhizal root systems (sink) to support these beneficial interactions [58]. It has been demonstrated that an increased C demand up-regulates photosynthetic activity [59,60]. Specifically, the export rate of the new C sink is linearly correlated to the utilization of triose phosphate for sucrose synthesis and loading rates into the phloem, increasing the Pi recycling rate when releasing Pi back to the chloroplast [61] and activating the regeneration of ribulose 1,5-bisphosphate (RuBP) in the Calvin–Benson cycle (CBC) [62]. The stimulation of photosynthesis is evidenced by increased triose export caused by enhanced Pi availability inducing the activity of the electron transport chain for the photophosphorylation of ATP and reductants and preventing over-reduction of photosystem I [63]. A higher ATP/ADP ratio enhances the activation of the Rubisco provided that there is high C demand from the sinks [61]. Kiers et al. [64] concluded that plants detect, discriminate, and reward the best fungal partners by providing more carbohydrates to the most cooperative fungal partners that transfer more significant nutrient resources. Host C is transferred to the fungus (sink) as sucrose or hexose across the hyphae or arbuscules in the root cortex and transported as glycogen or triacylglycerol through the extensive extraradical hyphae network [65]. Depending on the model plant and mycorrhizal symbionts species combinations and the different culture conditions, previous studies showed that inoculated plants have lower levels of sucrose, glucose, and fructose in their source leaves, consistent with higher [66,67] or lower [68,69,70] sugar export. Mycorrhizal colonization of the host plant increases the expression of several intercellular sugars transporters (monosaccharide transporters (MSTs), sucrose transporters (SUTs or SUCs), and hexose and sucrose transporters (SWEETs)) in leaves and roots to increase the sink strength and thus unload more sucrose from the phloem [67,71]. These transporters are key components of C partitioning from source to sink organs (for reviews of phloem loading strategies, see [72,73,74,75,76,77,78]). For a review of the C metabolism in microorganisms, see [79]; MacLean et al., (2017) [80]. A large proportion of sugar is converted to fatty acids—the main C store—in AMF before being converted back (ca. 50%) to hexose to be used by fungi in the extraradical mycelium [58,79]. At the root level, before the transfer of hexoses at the plant–fungal interface, sucrose cleaving enzymes (sucrose synthase (SuSy) and/or invertases (Inv)) up-regulate their expression and activities in the presence of symbionts [67,70]. Despite the sugar transportome in beneficial plant–microorganism interactions, studies have shown that plants transfer lipids to symbionts to sustain colonization [80]. This transfer is first accompanied by the up-regulation of several genes encoding different fatty acid biosynthesis enzymes, such as pyruvate kinase, enoyl-acyl carrier protein (ACP) reductase I, and acyl-ACP thioesterase B [81,82]. In exchange, the AM fungi extraradical mycelium improves P acquisition by plants [83,84,85,86]; this P is used for energy supply, regeneration of the CO_2_ acceptor RuBP, and regulating the ratio of starch: Sucrose biosynthesis [87,88,89]. Moreover, mycorrhizal hyphae are thought to take up ammonium, nitrate, amino acids, and phosphate from the soil solution [90,91]. It should also be noted that there is a net transfer of C between plants linked by ectomycorrhizal hyphal networks [92], suggesting that the direction of C transport in mycorrhizas might be reversible.

Several studies on legume–rhizobia symbiosis, such as that in PGPR, demonstrated the importance of reduced C from plant photosynthesis in the form of carbohydrates, as well as amino acid and organic acid resources for the symbiotic N_2_-fixation that occurs in the root nodules [93,94]. The plant–microbe interactions may involve PGPR-triggered changes in plant sugar transport and C and N metabolism, which could lead to a regulated pool of sugars available for the PGPR and contribute to establishing and maintaining plant–PGPR symbiosis and its positive effects on plant fitness [95,96]. Root nodules are considered another large sink of photosynthetic energy by consuming ca. 10% of the plant’s photosynthetic output for N_2_ fixation. Therefore, N_2_ fixation in rhizobium–legume symbiosis is presumed to be limited by the amount of plant-derived photosynthate available to bacteroids, suggesting that increasing the ability of the endosymbiont to utilize photosynthate in the nodule may lead to increased N_2_ fixation rates. As N_2_-fixation enhances the leaf N mass fraction, it should also stimulate the plant photosynthesis rate by increasing RuBP activity and electron transport rates [97]. The N mass fraction in the leaves and the rates of the photosynthesis relationship are not consistently linear since the net photosynthesis rate follows a linear-plateau response to N_store_ and may not increase above a threshold of leaf N sufficiency (ca. 2% on a dry weight basis) [98,99]. Sucrose, transported from the shoots to nodules, is the primary C resource for energy supply and C skeletons for symbiotic nitrogen fixation. This sucrose is then processed by enzymes, such as SuSy and alkaline invertase, to cleave the sucrose into substrates (i.e., UDP-glucose and fructose/glucose) to be used by bacteroids to support nitrogenase activity. The resulting free hexoses are further metabolized to dicarboxylates, particularly malate and succinate—the forms provided by the host plants for bacteroid activities (For a review, see [100]). Sucrose metabolism is essential for the development, function, and efficiency of SNF (symbiotic nitrogen fixation), as sucrose synthase was proven to be essential for SNF in plant nodules [101]. Colebatch et al. [102] showed that several metabolic pathways, such as those for CO_2_ fixation, glycolysis, and amino acid biosynthesis, appear to be coordinately upregulated in nodules. In the other counterpart, the nitrogenase enzyme complex is the central unit of N fixation in the bacteroids. The produced NH_4_^+^ is released into the cytosol of the infected host cells, where ammonia is converted first to glutamine and glutamate. These are then further converted to aspartate and asparagine in indeterminate nodules or ureides in determinate nodules as the final products exported into the host plant (For a review, see [99]). It was shown that when successful symbiosis is established, biological N_2_-fixation can supply the majority of the N required by host plants [100]. Flux balance analysis and a series of simulations using a “Virtual Nodule Environment” (ViNE) model of a genome-scale metabolic network in the SNF suggested that the metabolic costs associated with symbiotic N fixation are primarily related to supporting nitrogenase activity, and increasing N_2_-fixation efficiency is linked with decreasing returns in terms of the rate of plant growth [103].

In contrast to the situation in mutualistic symbiosis, biotrophic pathogens, including fungi, bacteria, and viruses, hijack host cells plants to suppress host immunity and take advantage of their nutrients, mainly sugars, for surviving and reproducing without returning any benefits to the host. The interactions between plants and biotrophic fungi (e.g., rust and powdery mildew) are cited more often as models for the study of pathogen-related modifications of C and N metabolism and partitioning. Sutton et al.’s [104] experiments on the uptake of C-derived metabolites and the competition between sugars in wheat-powdery mildew association showed that sucrose is hydrolyzed before uptake in that system. The induced expression of cell wall Inv, SUTs, and SWEETS during beneficial microbial interactions has similarly been targeted by plant pathogens to acquire sugars for their growth [67,105]. Zhao et al. [106] reported that sucrose is hydrolyzed into glucose and fructose, which are then taken up by the bacterial hexose carrier. In response, the plant cell retrieves the hexoses through a hexoses carrier to starve the pathogen. The retrieved glucose and fructose may play a signaling role and activate the antioxidants (e.g., glutathione and ascorbic acid) and defense molecules (e.g., salicylic acid and callose). Further, the localization of the sugar facilitator SWEET to the tonoplasts of the root cells could play an essential role in vacuolar glucose sequestration, thereby modulating the availability of sugars and limiting the secretion of C from roots [107]. These bacteria, as a countermeasure, may produce decoy molecules, such as trehalose, to fool or disrupt the host sensing and defense mechanism.

In the same line, plant–biotrophic virus interactions cause profound and dynamic modulations of the host’s primary metabolism. These perturbations include soluble sugar and starch accumulation at the infection sites, as well as reduced photosynthetic capacity indicative of a source to sink conversion [108]. Previous studies showed that plasmodesmata act as important control sites for carbohydrate transport and allocation as part of the metabolic consequences of viral infections in plants [109,110]. Indeed, infected plants showed dilated plasmodesmata in parallel with significant modulation in photosynthetic capacity, C metabolism, and resource allocation influencing symplastic sucrose transport to the phloem [111,112]. Less et al. [113] observed downregulated transcripts associated with assimilatory processes such as photosynthesis, starch metabolism, lipid metabolism, C1 metabolism, and the biosynthesis of amino acids after treatment with virulent or avirulent pathogens or pathogen-derived elicitors.

Viruses exploit the assimilate transport system for their long-distance transport by interacting with the viral proteins to host factors and components of the long-distance transport machinery. In exchange, by activating the genes involved in carbohydrate catabolism cascades, such as glycolysis and the TCA cycle, plants use C and N metabolic pathways not only as a source of energy to drive extensive defense responses but also as a source of signaling molecules to trigger defense responses. N metabolism via the amino acids is also likely to influence the outcome of plant–pathogen interactions [114]. For information on other topics related to plant–pathogen interactions, readers may refer to specific articles covering the regulation of metabolism [115,116,117] and interacting machinery [118,119], as well as host-specificity factors and molecular characteristics [120,121,122,123].

Microbes also synthesize and emit many volatile compounds (VCs) with low molecular masses (<300 Da), high vapor pressure, and low polarity [124,125]. These VCs, as long-distance messengers, involve plants’ “primary” metabolic pathways in C and N metabolism. VCs may play potentially essential roles as semiochemicals in interspecies communication, participating in countless interactions among plants and microorganisms due to their evaporation and diffusion properties, both below- and above-ground [126]. VCs emitted by bacteria and fungi can exert either inhibitory or stimulatory effects on plant growth by targeting the biochemical reactions that take place into the living cells [127,128,129,130]. Microbial VCs are involved in both microbe–microbe and microbe–plant interactions. The roles of VCs in positive and antagonistic interactions within the microbial world occurring below-ground, such as bacteria–bacteria, fungi–fungi, fungi–bacteria, bacteria–protists, fungi–plant, bacteria–plant, and bacteria–fungi–plant interactions and their ecological significance, have been reviewed recently [131,132,133,134]. As an inhibitory effect, VCs function as suppressors of immune responses, likely leading to the direct or indirect involvement of reactive oxygen species (ROS) as a signal leading to chlorosis. It has also been described that some VCs, via the primary transcriptional response, negatively affect the biological membranes represented by transport systems for sugar and amino acid permeability [135]. Further, Stall et al. [136] showed that the ammonia produced by *Xanthomonas vesicatoria* can foment a necrotic formation in pepper leaves, likely after the leakage of nitrogenous materials from the protoplasm; bacterial cyanide and ethylene have also been shown to influence plant fitness negatively [137,138].

Conversely, depending on the microbial culture conditions, volatile emissions from some beneficial rhizosphere bacteria and fungi can promote plant growth [139,140,141,142,143]. Microbial VCs can promote changes in plants’ photosynthetic capacity, C metabolism, and transitions from source to sink status in photosynthetic tissues [144]. Furthermore, Ezquer et al. [145] and Li et al. [146] showed that VCs emitted from several microorganisms, ranging from Gram-negative and Gram-positive bacteria to different fungi, promote the accumulation of exceptionally high levels of starch in leaves of mono- and di-cotyledonous plants. The analysis of Arabidopsis mutants with perturbations in hormone production and signaling, in conjunction with an analyses of hormone contents, indicated that the accumulation of high levels of starch in leaves through cytokinin-regulated processes may participate in the growth-promoting effects of VCs, suggesting the involvement of complex signaling mechanisms [140]. Moreover, Arabidopsis plants can respond to the VCs emitted by phytopathogenic microorganisms by triggering plastidic phosphoglucose isomerase (pPGI)-independent mechanisms [141]. The pPGI—an enzyme with an essential role in connecting the CBC with the starch biosynthetic pathway—acts as an important determinant of photosynthesis and growth, likely as a consequence of its involvement in the synthesis of plastidic cytokinins (CKs) in roots. Indeed, the promotion of photosynthesis, growth, and starch over-accumulation by VCs stimulates pPGI-independent mechanisms as a consequence of the photosynthesis-driven enhancement of plastidic CK production in leaves, allowing further photosynthesis promotion. This phenomenon is accompanied by the activation of N metabolism through the accumulation of β-alanine and GABA amino acids [141]. Recent studies performing transcriptomic and redox-proteomic analyses identified posttranslational thiol redox proteome changes involving VC-responsive genes and/or signaling reactions that promote the reductive activation of photosynthesis-related proteins. This latter effect results in the augmentation of photosynthetic activity; this, in turn, enhances the synthesis of glyceraldehyde 3-phosphate (GAP), which then enters the plastidial methylerythritol 4-phosphate (MEP) pathway, fueling the production of isoprenoid hormones that initiate a cascade of redox-regulated signaling reactions, resulting in changes in the expression of genes whose translation is subject to redox regulation [142,143]. Moreover, microbial VCs have also been shown to increase root biomass and architecture, despite the aerial part [142,147,148]. These changes result from the signaling of enhanced photosynthetic CO_2_ fixation employing CBC intermediate(s) or their derivatives, such as GAP, which is the first point of regulation in the synthesis of isoprenoid compounds derived from the MEP pathway, including hormones, and the resulting changes in the expression of a significant number of CK-regulated genes [142].

## 4. Carbon and Nitrogen Metabolism under Environmental Stresses

The most remarkable end-product of carbohydrates fixated by photosynthesis is the starch in plastids. Starch is a characteristic storage substance in plants that is not synthesized in other organisms. Starch is also the most important carbohydrate in the human diet. Starch is composed of amylose and amylopectin, which are glucose homopolymers and appear as semi-crystalline granules in plastids. Amylose consists of long straight polymers with very few α-1,6-branch points. On the other hand, amylopectin is a highly branched α-1,4- and α-1,6-linked glucose polymer, which occupies 70–80% of starch granules in most plants. Amylopectin is involved in the formation of a semi-crystalline structure with crystalline and amorphous lamella, and amylose likely does not contribute to the starch granules’ structural formation [149].

**Figure 6:**
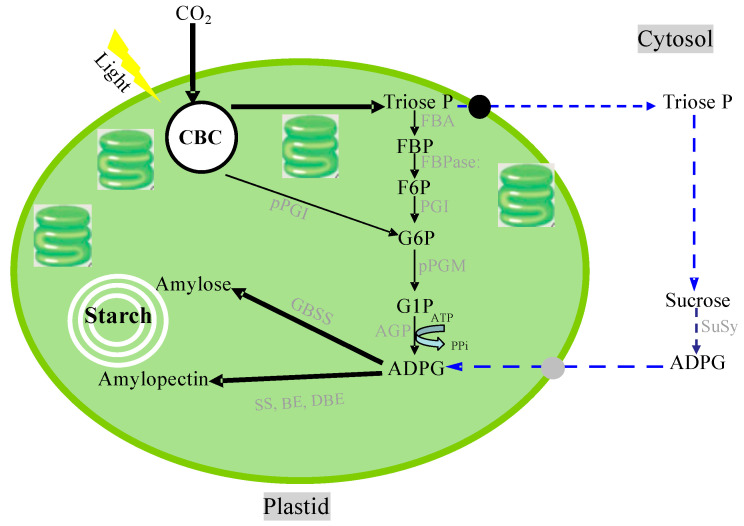
Starch biosynthesis pathway in leaf plastids according to the “Classic model” (black arrow) and alternative model (blue arrow; see [150,151]). CBC: Calvin–Benson cycle; FBA: Fructose-1,6-bisphosphate aldolase; FBPase: Fructose 1,6-bisphosphatase; PGI: Phosphoglucoisomerase; PGM: Phosphoglucomutase; SuSy: Sucrose synthase; GBSS: Granule bound starch synthase; SS: Starch synthase; BE: Starch branching enzyme; and DBE: Starch-debranching enzyme.

Starch biosynthesis is performed with at least four enzymes: ADP-glucose pyrophosphorylase (AGPase), starch synthase (SS), starch-branching enzyme (BE), and starch-debranching enzyme (DBE). However, there are several proposed pathways for starch biosynthesis in plastids (Figure 6). Indeed, an increasing volume of evidence has been provided recently that supports the occurrence of additional/alternative starch biosynthetic pathways involving the cytosolic and plastidial compartments wherein the supply of ADP-glucose (ADPG) is not directly linked to the CBC by means of pPGI [150,151]. ADPG is the glucose donor for the α-glucan elongation of starch and is formed from ATP and glucose 1-phosphate by AGPase. SS catalyzes the transfer of the glucose moiety from ADP-glucose to the non-reducing end of a-glucan for the elongation of amylose and amylopectin molecules. BE catalyzes the hydrolysis of an a-1,4-linkage and the subsequent transfer of a-glucan to form an a-1,6 branching point. DBEs, namely isoamylases, are involved in the formation of the highly ordered amylopectin in vivo to build semi-crystalline starch granules. Plastids, including chloroplasts in green leaves and amyloplasts in starchy cells, contain the genetic machinery required to synthesize their own proteins. About 100 proteins are encoded by the chloroplast genome [152,153]; however, 2000–3000 nuclear-encoded preproteins are found in the chloroplast [154,155]. All starch synthesizing enzymes are encoded in the nuclear DNA. Nuclear-encoded plastidial proteins are generally synthesized in the cytosol and post-translationally imported into the organelle. The precursor proteins of starch synthesizing enzymes possess an N-terminal presequence called a transit peptide [156,157,158,159], which is necessary for, and also sufficient for, plastidial targeting and translocation initiation. The transit peptide of granule-bound starch synthase I (GBSSI) (*Waxy*) has been investigated and characterized, indicating that the structural features of transit peptides are conserved in both monocot and dicot plants [160,161,162]. In rice, barley, maize, potato, and pea, the transit peptides with lengths of ca. 80 amino acid residues exhibit typical properties of chloroplast transit peptides, which are rich in hydroxylated (Ser, Thr), hydrophobic (Ala), and positively charged (Arg, Lys) amino acids and deficient in acidic amino acids; further, positively charged amino acid residues are rarely found within the 15 N-terminal regions of transit peptides [157]. The transit peptide is caught and interacts with the translocon at the outer envelope of the chloroplast (TOC) complex; then, protein import across the inner envelope is facilitated by the translocon at the inner envelope of chloroplast (TIC) complex [163,164,165]. After import, processing and folding of the precursor protein take place inside the plastid.

Starch biosynthesis is regulated at both the transcriptional and posttranslational levels. Transcriptional regulation provides a long-term adjustment of starch biosynthesis. Lights regulate the expression of many genes, including chloroplast biogenesis, chloroplast gene expression, and photosynthesis-associated processes, such as chloroplast movements [166,167]. There exist more than 40 TFs that act downstream of photoreceptor genes in Arabidopsis [168]. The TFs regulating starch biosynthesis have been also identified in rice [169,170,171,172,173,174], maize [175,176,177,178], barley [179], cassava [180,181], *Panicum virgatum* [182], sweet potato [183], and Arabidopsis [184,185,186,187]. It is noteworthy that a rice nuclear factor Y (NF-Y) TF complex activates GBSSI to regulate the grain quality of rice [188].

Posttranslational modifications (PTMs) are recognized to be the main way by which the enzyme activities involved in transient starch metabolism are regulated [189,190]. Several starch metabolism-related enzymes can be reduced and activated by thioredoxins and NADP-dependent thioredoxin reductase C (NTRC). AGPase has been especially well-studied and characterized in potato [191,192], pea [193], Arabidopsis [194,195], and rice [196]. AGPase is allosterically activated by 3-PGA and inhibited by P_i_. The activity of the plastid-localized enzyme is also regulated by redox control in response to environmental changes. The less-active oxidized form contains an inter-subunit disulfide bond formed between the pair of small subunit Cys12 residues of the heterotetrameric enzyme. In rice, the major endosperm AGPase demonstrated that the cytosolic isoform, like plastidial enzymes, is subject to redox control. Cysteine residues at the N-terminal of the large subunits C47 and C58, but not C12, were shown to be essential for the proper redox responses of the enzyme [196].

Starch degradation has been intensively investigated in germinating cereal seeds; α-amylase, debranching enzyme (R-enzyme), and α-glucosidase are involved in the breakdown process [197,198]. Recently, the degradation of transient starch in the chloroplast was clarified in Arabidopsis [149,199,200]. The starch molecules are phosphorylated and dephosphorylated by glucan water dikinase (GWD), phosphoglucan water dikinase (PWD), and phosphoglucan phosphatase (DSP). This phosphorylation–dephosphorylation conversion is essential for normal starch degradation [201,202]. Starch is broken down into maltose and glucose by the coordinated action of β-amylase (BMY), isoamylase (ISA), and disproportionating enzyme (DPE) [203,204]. In Arabidopsis chloroplasts, α-amylase (AMY) is involved in most minor pathways of starch degradation [205].

Plastid starch degrading enzymes are also encoded by nuclear genome DNA. Various features of transit peptides among Arabidopsis enzymes were also observed. BMY8, DPE1, phosphorylase (PHS1), GWD1, ISA3, DSP4, and AMY3 have transit peptides with lengths of 30 to 83 amino acid residues. Although these transit peptides are commonly rich in Ser, both positively and negatively charged amino acid residues are frequently found within the N-terminal region, unlike the canonical features of transit peptides [206]. Furthermore, a recent investigation revealed that tissue-specific transit peptide motifs function in isolated pea (*Pisum sativum*) leaf chloroplasts and root leucoplasts [207]. Thus, the TOC–TIC import machinery should be diverse to accept many types of transit peptides.

It has also been shown that α-amylase significantly contributes to the mechanism of starch breakdown in living cells of cereals [208,209,210]. The polymorphic enzymes α-amylase AmyI-1 (*Amy1A*), II-3 (*Amy3E*), II-4 (*Amy3D*), and II-5/6 (*Amy3B/3C*) are extensively expressed in rice plants and tissue cultures [211]. The sequence alignment of these isoforms revealed that the presence of the N-terminal signal peptide for ER membrane translocation is present in all precursor forms of these isoforms, but no transit peptide was found [212]. Studies using transgenic rice plants with suppressed expression or overexpression of AmyI-1, immunocytochemical analysis with specific anti-AmyI-1 antibodies, the expression and targeting of AmyI-1-GFP in redifferentiated green cells, and the cell biochemical analysis of chloroplastic AmyI-1 demonstrated that AmyI-1 is present in the chloroplasts of rice [209,210,212]. The imaging analyses showed that the plastid targeting of AmyI-1 was inhibited by both dominantly negative and constitutively active mutants of Arabidopsis ARF1 and SAR1, which arrest endoplasmic reticulum-to-Golgi traffic. Contact of the Golgi-derived membrane vesicles with the cargo and subsequent absorption into plastids occur, as observed by employing three-dimensional time-lapse imaging and electron microscopy of high-pressure frozen/freeze-substituted cells. Thus, it is evident that Golgi-to-plastid traffic is involved in the plastid targeting of AmyI-1. Furthermore, the experimental results of the transient expression of a series of C-terminal-truncated and site-directed mutated AmyI-1-GFP fusion proteins suggested that multiple surface regions, including the putative starch binding site Typ-301–Trp-302, are necessary for plastid targeting. This plastid targeting seems to be accomplished in a sorting signal-dependent manner [212]. Moreover, rice long-chain acyl-CoA synthetase 9 (OsLACS9) in the chloroplast envelope membrane was suggested to be involved in the plastid localization of AmyI-1 using the *lacs9* mutant line [213]. However, the mechanism for importing vesicles trafficking with nucleus-encoded proteins into plastids is a black box [214]. Notably, starch metabolism-related enzymes transport, localize, and functionalize plastids via diverse routes [215]. Diversification of the starch biosynthesis pathways occurring in both autotrophic and heterotrophic organs is likely finely regulated in response to the environmental inputs.

Climate variations, global warming, and the related impacts, such as drought, sea-level rises, typhoons, and soil salinity, significantly damage agricultural activities worldwide. Recently, extremely high temperatures during the ripening season of rice caused a decrease in yield and grain quality. It was reported that heat stress causes and stimulates grain chalkiness, which refers to damaged grain with loosely packed abnormal starch granules [216,217,218,219,220]. Severe grain chalkiness can lead to reductions in grain weight, resulting in yield losses.

Although normal translucent grains of rice are filled tightly with polygonal granules, the chalky zone of the grain contains round starch granules with numerous air spaces between them [221]. This phenomenon suggests that starch metabolism in the developing endosperm is disordered under high temperatures. Transcriptomic analyses of ripening seeds showed that the key enzyme genes for the synthesis of amylose and amylopectin, granule-bound starch synthase (*GBSSI*), and starch branching enzyme (*BEIIb*) are downregulated under high-temperature conditions, leading to low contents of amylose and long-chain-enriched amylopectin in mature grains [222]. The decrease in GBSSI protein was confirmed by several proteome analyses [223,224], and the activities of GBSSI and BEIIb were reduced at elevated temperatures [225,226]. Moreover, the other genes related to starch synthesis, including ADP-glucose pyrophosphorylase, ADP-glucose translocator, and SuSy, were also downregulated under high-temperature stress [222].

In contrast, the expression of starch-hydrolyzing enzymes, a series of α-amylases, as well as their enzyme activity, were dramatically increased by heat stress, strongly suggesting that starch degradation occurs in the developing endosperm under stress conditions [222,224,227,228]. Thus, it is conceivable that starch accumulation and granule formation are achieved through the turnover of starch via biosynthesis and degradation and that high temperatures impair the formation of starch granules through a combination of decreased biosynthesis and increased degradation. A detailed analysis of a tissue-specific transcriptome employing the laser microdissection technique suggested that different areas of the endosperm tissue, designated as dorsal, central, and lateral tissues, exhibit individual responses to heat stress [229]. It appears that the differential and characteristic expression of heat shock proteins is involved in controlling redox, N, and amino acid metabolism in the endosperm, which is possibly linked to grain chalking under heat stress [229].

Metabolomic analysis provided suggestive information concerning the effects of high temperatures on the grain filling of rice. The contents of sugar phosphates related to glycolysis and intermediates of the citric acid cycle decreased, while those of amino acids increased in developing seeds under elevated temperatures [230]. It is well-known that factors other than starch metabolism are also involved in the regulation of grain size and starch quality [231,232]. Genes of both *GLUTELIN PRECURSOR MUTANT 6* (*GLUP6*) and *GLUTELIN PRECURSOR ACCUMULATION 3* (*GAP3*) are related to the accumulation and formation of protein storage organelles in rice. *GLUP6* is a guanine nucleotide exchange factor involved in intracellular transport from the Golgi apparatus to the protein storage vacuole, and *glup6* mutants accumulate an abnormally large amount of proglutelin [231]. The *GAP3* gene is involved in post-Golgi vesicular traffic for vacuolar protein sorting [232]. The mechanism of grain chalkiness caused by high-temperature stress can be highly complex.

Intracellular and extracellular proteins are subjected to proteolysis, in which the balance between protein synthesis and protein turnover should be maintained. Two main processes are responsible for intracellular protein turnover: The ubiquitin–proteasome system and autophagy. The former is considered the most extensive protein disposal system [233,234], and in the later, the proteins are targeted to the lysosomes for degradation via the action of enzymes [235,236]. Autophagy is an evolutionarily conserved intracellular destructive mechanism that degrades intracellular proteins, metabolites, and intracellular organelles for recycling and quality control [237] and plays essential roles in C and N metabolism, as well as the growth, development, and survival of eukaryotic cells [238,239,240]. Furthermore, a recent investigation demonstrated that autophagy deficiency in rice leads to a chalky appearance of grains, even under normal growth condition [241]. The rice autophagy-deficient mutant *Osatg7-1*, which produces seeds at a shallow frequency in paddy fields, exhibited a chalky appearance and lower starch content in the endosperm of mature grains. Electron probe microanalyzer (EPMA) images showed small pits on the surfaces of the starch granules from the chalky zone of the *Osatg7-1* grain. A comprehensive analysis of changes in the proteome and the biochemical properties of the *Osatg7-1* grain found the abnormal activation of starch degradation pathways, including the accumulation of α-amylases in the endosperm during seed maturation in *Osatg7-1*. Interestingly, these phenotypes of the *Osatg7-1* seeds closely resembled those of the wild type seeds exposed to elevated temperatures during ripening. This provides insights into the novel autophagy-mediated regulation of starch metabolism in the endosperm under heat stress during seed maturation.

**Figure 7:**
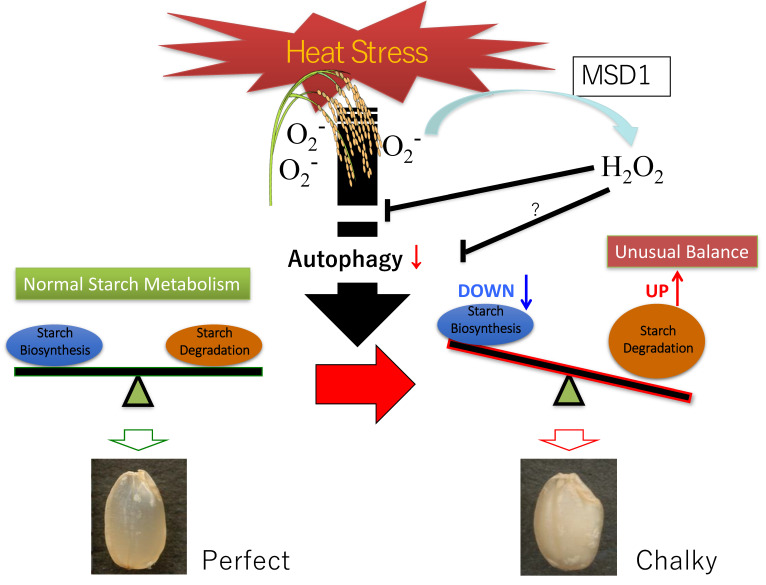
A proposed model of grain chalking under high-temperature stress. MSD1: Mn-type superoxide dismutase 1.

A proposed model of grain chalking under high-temperature stress is illustrated in Figure 7. High-temperature ripening causes an unusual balance in the synthesis and degradation of starch in the endosperm tissue; hence, this ripening could be considered the leading cause of chalkiness formation. On the other hand, high-temperature stress also dramatically altered the metabolism of proteins and amino acids [230]. There is no doubt that the recycling and quality control of proteins are critical for perfect and healthy starch accumulation in the developing endosperm of rice. Autophagy may also play significant roles in the regulation of starch and sugar metabolism, as well as environmental stress adaptations, to, e.g., heat, during seed maturation. The overexpression of superoxide dismutase (MSD1) significantly improved heat stress tolerance after heading [242]. The prompt enhancement of the H_2_O_2_ level by MSD1 under high-temperature stress is also likely essential. It has been recently reported that autophagy controls ROS homeostasis in guard cells and is essential for stomatal opening in plant leaves [243]. Notably, H_2_O_2_ treatment was shown to alter and promote autophagic flux in mammalian cells [244,245]. We infer that there is possible involvement of H_2_O_2_ signaling and autophagic control in normal starch granule formation and accumulation in the grain filling stage of rice. These findings shed light on a possible novel strategy for the protection of grain quality and crop yield affected by environmental stresses [241].

## 5. (Multi)-omics Techniques to Study C and N Metabolism in Plants

As reviewed above (Section 1 and Section 2), central C and N metabolism and regulation have been addressed, and information is available to generate a highly complex network that describes their metabolic and regulatory interactions. Research is focusing on understanding the natural variation within a species that fine-tunes these processes in individual plants, as well as the key genes, proteins, and metabolites involved in such processes. To visualize the different types of interactions and the conciliatory, regulatory, and structural networks is of great interest to decompose the various structural and functional components, from gene expression to translation and from activities to fluxes. The analysis and integration of (multi)-omics can uncover the cellular responses to stimuli or the mechanisms of action of the metabolic pathway at a system level. Qualitative and quantitative systems biology uses a holistic and integrative approach to understand the whole process, from cell to community levels, related to growth, development, and environmental adaptation. Omics approaches (e.g., (meta)-genomics, transcriptomics, proteomics, and metabolomics) provide a more holistic molecular perspective of central C and N metabolism, which involves a set of coordinated movements that supply molecules, energy, and redox power to the plant cell to sustain survival, growth, and adaptation.

### 5.1. Genomics

Recent ongoing (meta)-genomics and single-cell techniques have given researchers a glimpse into the genomic information and genetic diversity of C and N metabolism. Studies have identified a large number of quantitative trait loci (QTL) to dissect C and N metabolism in many species [246,247,248,249]. These seminal works revealed the key genetic regions underlying variations in core C and N metabolism, such as plant photosynthesis and NUE [250]. These genetic regions include candidate genes known to be involved in C and N processes. Results from nested association mapping in a 282 inbred association panel using both genome-wide association study (GWAS) and candidate gene association approaches identified many beneficial alleles related to C and N metabolism that will be useful for improving kernel starch, protein, and oil content [251]. Zhang et al. (2015) [250] used GWAS in maize to identify the associations of many genes involved in C and N metabolism. Liu et al. (2016) [252] used GWAS to uncover the SNPs, QTLs, and candidate genes that elucidate the genetic basis for starch content that could be used for improving germplasm via marker-assisted selection in breeding. The candidate genes identified in these studies are good targets for improving C and N metabolism in maize and other crops and may help determine the molecular mechanisms regulating the relevant metabolic processes. However, studies on genetic variation in C and N metabolism are limited because these works identified trait loci only through linkage mapping in artificial families or through association mapping across populations of unrelated individuals [250].

Additionally, many of these studies focused on C and N in controlled experiments under greenhouses or growth chambers instead of under field conditions, risking the omission of critical genetic loci if the conditions do not include important natural environmental variables [250]. Recently, advancements in cutting-edge genome-editing technologies, i.e., the clustered regularly interspaced short palindromic repeats (CRISPR)/CRISPR-associated protein 9 (CRISPR/Cas9) system, have been used for genome editing in several species targeting C and N metabolism [253,254,255,256]. Indeed, the authors in [257] investigated the importance of an individual enzyme in the CBC, sedoheptulose-1,7-bisphosphatase (SBPase: EC 3.1.3.37), in altering photosynthetic capacity and carbohydrate accumulation, as well as changes in the levels of protein and amino acids and activities of N metabolic enzymes.

### 5.2. Transcriptomics

The metabolism functions of C and N are closely intertwined and need to be tightly coordinated to maintain a balance between C and N metabolites in plants This type of omics enables the identification of genes and TFs physiologically involved in the interactions between the C and N metabolic pathways, as well as the mechanism for sensing and regulating C:N (im)balance. A microarray analysis was used to study global changes in mRNA levels, revealing over 300 genes to be deregulated in *Arabidopsis* seedlings subjected to combined C:N treatments compared to C or N treatments, thus providing in vivo evidence supporting the hypothesis that plants have a carbon/nitrogen-sensing/regulatory mechanism [258]. Huang et al. (2016) [259] identified several functionally correlated genes (chalcone synthase, chlorophyll a-b binding protein, oxidase 1B, malate dehydrogenase, and lysine and histidine specific transporter 1) that are responsive to imbalanced C:N treatments in the aerial parts of rice seedlings and thus might control the average growth and development of plants. Since N status was found to be influenced by C status [260,261], other studies have used microarray technology to examine plant responses to N applications. For instance, *Arabidopsis* grown with several N sources showed a change in the expression levels of ca. 10% of the total detectable mRNA, with many of the altered genes involved in C and nutrient metabolism [31].

Moreover, microarray analysis was used to identify N-responsive genes because much less is known about the mechanisms controlling N regulation [30]. Kang et al. (2003) [262] proposed that glutamate receptor 1.1 functions as a regulator of C and N metabolism in *Arabidopsis*. Pérez–Delgado et al. (2016) [263] demonstrated several significant transcriptomic changes with possible interconnections between primary N assimilation and photorespiration that occur in the leaves of *L. japonicus* grown with different forms of N nutrition, including genes involved in N, C, and secondary metabolism, as well as TFs that could be involved in N signaling or metabolism. Similarly, other works detected transcriptome changes following different N-fed regimes [264,265,266,267,268]. Reports with microarray studies measuring the effects of a transgene on the plant transcriptome have also been carried out, thereby elucidating several genes, including CK-synthesizing adenosine phosphate-isopentenyltransferase (IPT), downstream genes of DREB1A (Dehydration-responsive element-binding protein 1A) (e.g., the C_2_H_2_-type zinc-finger motif and sugar transport protein), nitrate reductase (NIA2), GS, and GOGAT, that may play a role in N assimilation and regulation and the NUE phenotype, as well as shed light into C and N metabolism [269,270,271,272].

Although microarray studies have been used for the past few decades, the use of RNA-Seq is more recent and can precisely measure transcript levels, allowing for the absolute quantification of variations in gene expression of C and N metabolism [273]. Recent works employed RNA-Seq to investigate the responses of several C- and N-metabolism genes. The authors in [274] identified several differentially expressed target genes related to C and N metabolism after N-starvation, thereby allowing investigations into the signal transduction pathway of N-utilization. The analysis of rice and wheat transcriptomes using this technique showed the C metabolism of gene expression to be closely related to the level of N supply and environmental conditions [275,276]. These results identified several candidate genes that are differentially expressed, including protein kinases, receptor kinases, and TFs. The authors also suggested the reasonable regulation of C and N metabolism as a way to improve NUE [275] and the reprogramming of primary and secondary metabolism under the future climatic scenario [276]. The study in [277] was carried out to identify the molecular networks of C and N metabolism in cotton. These authors identified several hub genes that might provide novel insights into coordinating C and N metabolism, which could serve as the basis for high NUE. Moreover, Li et al. [278], using whole-genome RNAseq and small RNAseq analyses, studied how the natural light/dark cycle regulates C and N metabolism to ensure plant growth and development. The results revealed differentially expressed genes and miRNAs involved in the C and N metabolic pathways that mediate most instances of post-transcriptional regulation in response to environmental changes. Altogether, these studies indicate that the reasonable regulation of C and N metabolism could provide an effective way to increase crop yield and reduce environmental costs. At the cellular level, Gifford et al. (2008) [279] showed the distinct cellular responses of five Arabidopsis root cell types to N influx, such as the cell-specific regulation of hormone signaling. The authors in [280,281] focused on how single-cell RNA-seq could have the potential to generate datasets, including C- and N-related components, to resolve the molecular relationships among individual plant cells and determine plant tissue organization, developmental dynamics, and physiological responses. These result sets require integration with one another and also ideally with targeted functional genomic studies, such as the establishment of the gene network controlling both type of metabolism to obtain a comprehensive overview of the system biology of the plant cell.

### 5.3. Proteomics

Understanding the proteomic traits of C and N metabolism could elucidate novel molecular targets, whose intervention may lead to improvements in crops. Despite its present high costs and inability to cover all proteins/cell types, this type of omics provides direct evidence for the presence/absence of proteins or enzymes or the intricate mechanisms that coordinate and control the pathways involved in the sensing and acquisition of C and N. In this context, proteomics studies are useful in deciphering new aspects of the interlinks between C and N metabolism as one of the leading sinks for the reduction in power produced by the light reactions of photosynthesis [282,283,284]. Indeed, the C fixation and assimilation of leaves are related to N content primarily because the proteins of the CBC and thylakoids represent the majority of leaf N. In recent decades, 2-D gel electrophoresis (2-DE) combined with mass spectrometry (MS) has been widely utilized to detect correlations between differential protein expression and plant C–N interactions in various tissues of rice, wheat, maize, and other species. However, these techniques are scarcely used due to their drawbacks related with their low resolution and limited samples. Recently, advanced quantitative proteomic techniques have improved the coverage of total (sub)-proteomes and have characterized signaling pathway(s) and PTMs, as well as protein–protein interactions at the cellular level, shedding light on the organ- and tissue-specific mechanisms of C and N metabolism as a response to environmental fluctuations during the plant life cycle.

Quantitative proteomic methods along with label-based and label-free MS-based approaches (e.g., tandem mass tags (TMT) and isobaric tags for relative and absolute quantification (iTRAQ)) are more precise and efficient approaches to achieve multiplex quantitation without increasing spectral complexity. By applying the 2-D gels of leaf proteins at the vegetative stage of maize plants grown under high or low N supply, Amiour et al. [284] revealed a lower amount of the ribose-5-phosphate isomerase precursor that would eventually produce a functional enzyme of the CBC and an increase in the level of glycolytic enzymes such as enolase, fructose-bisphosphatase, aldolase, and fructose 1,6-bisphosphatase. These enzymes are involved in facilitating efficient C assimilation and the closely related metabolic pathways. This investigation and others [285,286] confirmed that the metabolism of both C and N is intertwined, along with the energy requirement of C and N at the proteomic level. Comparative proteomics studies showed that NO_3_^−^ availability evokes different responses in roots and leaves [287] and induces changes in the protein abundances involved in N (e.g., amino acids metabolism in both leaves and roots) and C (e.g., photosystems proteins and glycolytic enzymes) metabolism [287]. Chandna and Ahmad [288] provided new insights into the altered protein patterns related to photosynthesis (e.g., RuBP, chlorophyll-protein complex, and photosynthetic C reduction cycle), glycolysis (fructose-bisphosphate aldolase and cytosolic glyceraldehyde-3-phosphate dehydrogenase), and N metabolism (nitrate reductase, glutamine synthetase, and porphobilinogen deaminase) in response to the N supply. These data suggested that genes functioning in many physiological events coordinate the responses to the availability of N and the improvement of the NUE of crops. The iTRAQ approach was applied to deeply study, among others, the underlying mechanisms of stress responses since the coordination of C and N metabolism is essential for plants to adapt to fluctuating conditions. iTRAQ was successfully applied to conduct a comparative rice grain proteomic analysis during grain filling under moderate and high-temperature stress [224]. Similarly, Inomata et al. (2018) [289], using (phospho)-proteomics, revealed the uncontrolled activation of photosynthesis and protein synthesis after a mutation in the nucleotide pyrophosphatase/phosphodiesterase 1 (NPP1) gene of rice, which controls C flux by transporting C taken up from starch and cell wall polysaccharide biosynthesis to other metabolic pathways, under high-temperature and high CO_2_ conditions. Moreover, iTRAQ can address the specific biological involvement of C and N metabolism in defense analysis. For instance, Ma et al. (2019) [290] revealed the reduction of C- and N-metabolism to be involved in the red-skin disorder mechanisms affecting ginseng plant growth, thereby impairing the quality and yield and impeding continuous cropping.

Subcellular proteomic analysis has been proven to be a powerful technique for refining our knowledge of the cellular process network in one particular organelle, including the chloroplast, mitochondria, nuclear, or plasma membranes [153,291,292]. For instance, proteomic studies of wheat and soybean chloroplasts have contributed to a better understanding of the metabolic process through which the identified proteins involved in antioxidant defense and C metabolism directly help C become incorporated into organic compounds [293,294,295]. In addition, Tejada–Jimenez et al. [296] showed that chloroplast signals are needed for the synthesis of nitrate and nitrite reduction, which are necessary for optimal N assimilation, since the chloroplast contains up to 75% of leaf N in the form of Rubisco components [297]. Mitochondrial proteome analyses in rice and wheat have provided valuable molecular insights into both the individual proteins and the protein complexes that participate in salinity response mechanisms [298,299]. These proteins are linked to the mitochondrial enzymes of C and N metabolism and are a key mediator of salinity tolerance since mitochondrial processes regulate the accumulation of N-derived osmolytes, such as GABA, proline, glycine-betaine, and the portion of fixed C that is allocated to respiration. Research on the cell wall proteomics of rice, soybean, and maize have given us a comprehensive understanding of how the cell wall protein composition changes in association with differential growth responses [300,301]. These results identified stress-responsive proteomes categorized into groups including carbohydrate and N metabolism with potential functions in the cell wall. A gel-free nano-LC MS/MS analysis of nuclear proteins changes under flooding stress in soybean root tips identified deregulated proteins related to C and N metabolism, such as the clathrin heavy chain, RACK1 (receptor for activated C-kinase 1) protein, and splicing factor PWI, including members of the serine/arginine-rich protein family [302].

At the root level, proteomic studies have focused mainly on understanding the impact of plant–microorganism interactions and functioning in C and N management (see Section 3). For instance, (phosphor)-proteomics studies have deciphered the phosphorylation-mediated signal transduction cascades, the complex network of kinase–substrate and phosphatase–substrate interactions in response to rhizobial infections, and the protein complex involving C and N metabolism in the mutual impact between microbes and host plants [303,304,305,306,307]. Furthermore, several proteomic studies have attempted to study how to balance root nitrogen gains with their energy needs and the developmental C costs [308], the C and N changes of the root system following symbiosis or pathogenesis infection [309,310], and the C–N complexity of constituents in root-to-leaf signaling [311]. In this root-to-leaf signaling, advanced studies on xylem and phloem proteomics have emerged to shed light on each plant fluid and the proteins associated with the developmental processes and biotic and abiotic stress responses involved in C and N functions. For reviews on this topic, readers are referred to [312,313,314,315].

At the leaf level, the proteomic approach has provided more in-depth insight into the C–N interconnection and enzymatic regulation in investigations related to (1) the regulation of chlorophyll biosynthesis and photosynthesis in crops [316,317,318]; (2) leaf senescence, mainly the investigation of N remobilization and the regulation of photosynthetic C metabolism, the importance of proteolysis, and chloroplast degradation [319,320,321]; and (3) sucrose and the glycolytic enzymes involved in its synthesis as the main photosynthetic product as a key component in C and N metabolism [319,322].

Comparative proteomic studies of the tissue-specific proteome in grains/seeds have been carried out to explore the expression of proteins and protein complexes involved in grain filling [323], the responses to stresses [324,325,326], the occurrence of chalky grains [224], and when seeds break dormancy and restart their metabolism to understand the role of C and N in the tissue-specific variation of metabolic proteomes in seed embryos and the surrounding tissues [327,328]. These studies provided insights into possible target proteins to adopt strategies against cellular activities to improve delayed seed germination and to better understand protein expression patterns along with their respective PTMs in grains for targeting crop seed quality under different environments.

### 5.4. Metabolomics

Metabolites, as the endpoint of gene–environment interactions, provide a more complete image of phenotypic differences. Metabolomics provides a sensitive and systemic technique to identify the alterations in endogenous low molecular weight compounds that reflect interventions in cellular physiology. The data obtained could be used to target subsequent up-stream proteomics/transcriptomics analyses to uncover the mechanistic proteins/genes driving C and N processes. The large-scale quantification of internal metabolites has been made possible thanks to developments in MS and NMR technologies. This could lead to a directed search for missing functions to generate new biological knowledge. Complex interactions via the biochemical networks of metabolite pathways participate in many aspects of C and N trade-offs. Thus, measuring metabolite levels using this omics technique provides necessary information about biological responses to the physiological or environmental changes triggered by C and N status [329,330]. Several studies have applied metabolomics technologies (i.e., GC-MS, LC-MS, CE-MS, GC-TOF/MS, Orbitrap-MS, FT-ICR-MS, and NMR) to assess metabolomic status and obtain insight into the network regulating C and N involved in a variety of metabolic events in several crops [331,332,333,334,335,336].

The (im)-balance of C fixation and N assimilation-derived metabolites has been suggested as the main factor responsible for plant growth and crop yields [337,338,339,340]. Numerous metabolomics studies have been published to address the role of the C and N metabolism of various plants tissues (i.e., root, leaf, grains, and fruits) (see the references herein) in “candidate” gene regulation and functioning [341,342,343,344], the responses of phenotypic changes [329], plant–microorganism interactions ([345,346,347], Section 3), plant stress responses ([348,349,350,351,352,353], Section 4), circadian clock functions [354], and improvements in crops and breeding materials [355,356]. Seminal studies have combined metabolite and transcript datasets through correlation and clustering analyses, further representing the datasets as connection networks between metabolites and genes and several plants to decipher the response mechanism(s) of rice to high night temperature [357], rice’s response to N nutrient supply [275], potato’s pigmentation mechanism [358], the blue flower formation mechanism in waterlily [359], and catechin production in the albino tea cultivar “YuJin-Xiang” [360]. Metabolomics, in combination with proteomics, has also helped decipher the complex molecular mechanisms underlying C and N traits. For instance, Wienkoop et al. [361] revealed several important metabolic (proline, glutamine, raffinose, and galactinol) and protein (GAPDH, cytosolic GAPDH, chloroplast chaperonin, and COR6.6) markers in *Arabidopsis* in response to cold or heat-induced stress. Kumar et al. [362] found efficient and increased C and N metabolism, the accumulation of phytoalexins, and lignification coupled with the enhanced accumulation of proteins related to pathogenesis in chickpea roots infected by *Fusarium oxysporum*. Similarly, Desalegn et al. [363] suggested the existence of an adjustment of C- and N-derived metabolites (TCA, amino acid, and secondary metabolism) and proteomes (proteins associated with pisatin biosynthesis) in response to pea plant metabolism under microbial symbiosis *Didymella pinodes* infection. Metabolomics has also been applied together with modelling for metabolites to predict reaction directionality.

Notably, the metabolome strategy can be untargeted, as a metabolite says nothing about the pathways in which it was synthesized or the fluxes that involve or target that metabolite when the metabolite is focused on the detection and quantification of specific classes of compounds or subsets of known metabolic pathways—for example, fluxomics, which measure intracellular fluxes using ^13^C or ^15^N labelled substrates to track each atom through the network (see [364,365,366,367,368,369,370]), and lipidomics, a subsection of metabolomics dedicated to lipid analysis, even if there is a continuum of polarity between lipophilic and hydrophilic metabolites (for reviews, see [371,372,373,374,375,376]).

Other omics studies are emerging, including, among others, peptidomics, glycomics, hormonomics, and phenomics. All represent a data type that can help determine the C and N cellular objectives that could have given rise to the phenotype.

The integration of omics data from multiple approaches represents a fundamental aspect of a systems strategy to provide a higher-order level of understanding of C and N metabolism from specific plant cell-types to the whole system. These omics technologies can obtain an integrated view of biological systems, thus bridging the genotype-to-phenotype gap to better define the phenotype. Indeed, combining C and N results from epigenomics and transcriptomics can determine the influence of cell-specific DNA modifications on gene activity. The integration of plant single-cell-type transcriptomes and proteomes provides the opportunity to better understand the regulation and dynamics of RNAs and their products in specific cells. Transcriptome–proteome studies could reveal the correlations between RNA-seq and proteomic data sets, as well as differences in, e.g., the rates of biosynthesis, degradation, and turnover of RNA and proteins. The integration of proteomics and metabolomics datasets could underpin the link between proteins/enzymes and their associated biochemical pathways, as well as their responses to environmental stimuli.

### 5.5. Metabolic Engineering in C and N Metabolism

C and N metabolic engineering aims to analyze and modify metabolic pathways to achieve some objectives related to, among others, improving yields, adapting to stresses, and facilitating the efficient production of industrially relevant compounds (See also [168,377,378]). Few significant improvements in C and N assimilation been achieved [379,380], due to the synchronous activation of a series of metabolic pathways that might be needed to influence assimilation. Additional attempts to change the C and N metabolism of plants for specific purposes have been carried out, starting with attempts to use chemical mutagenesis, such as N-ethyl-N-nitrosourea (ENU) or ethyl methanesulfonate (EMS), to speed up the selection and evolution processes in the desired direction. Previous results for lotus, whose mutants lack GS2 (Ljgln2-2), from lines subjected to EMS have shown the link between photorespiration, photosynthesis, and central metabolism to be tightly coordinated by Ljgln2-2 [381]. Other successful results of Arabidopsis metabolic engineering lines expressing maize TF DNA-binding with one finger 1 (Dof1) have shown increased N contents and growth rates under N starvation conditions [382]. Takahashi et al. [383] suggested that an increase in the biosynthesis of the cofactors (NAD and NADP) serving in numerous metabolic processes could also be used to modify C and N assimilation because high levels of a cofactor may stimulate multiple enzymatic reactions, resulting in synchronous metabolic pathway activation. However, these mutagenesis techniques struggled with the underlying mechanisms for the phenotype alteration, which made it difficult to identify relevant constraints and pathways. Recently, similar screens combining chemical mutagenesis and NGS (Next-Generation Sequencing) using approaches such as Mut-Seq, MutMap, and artMAP to probe for essential genes of phenotypes have been described in plants [384,385,386,387,388]. Subsequently, genetic modification has enabled the targeted modification of the C and N metabolic pathways that affect the metabolites that regulate and decide cell fate, organ physiology, and crop quality. For instance, the establishment of interlinks between glycolysis, sucrose metabolism, and organic acid biosynthesis was observed from transgenic plants expressing malate dehydrogenase (RNAi-mMDH) in tomato [389]. Studies silencing l-galactono-1,4-lactone dehydrogenase (Gal-LDH) or GDP-D-mannose 3,5-epimerase (GME), both enzymes of ascorbate biosynthesis, substantially altered cell size, respiration, photosynthesis, and fruit metabolites, thereby affecting fruit development [390,391,392].

Plants are continuously being challenged by the world around them, as they are involved in a complex network of interactions with microorganisms that influence their C and N partitioning. The relevant complex regulatory networks allow the integration of physiological and metabolic changes and the adjustment of C and N metabolism in the whole plant for normal growth and development.

## Data Availability

Not applicable.

## Abbreviations

3-PGA: 3-phosphoglycerate; AA: Amino acid; ABA: Abscisic acid; AMF: Arbuscular mycorrhizal fungi; AMT: Ammonium transporter; Aux: Auxin; CBC: Calvin–Benson Cycle; CEP: Carboxy-terminally encoded peptide; CEPD: CEP downstream; CLE: CLAVATA3-like; Cyt: cytokinin; GAP: glyceraldehyde 3-phosphate; Gln: Glutamine; Glu: Glutamate; GS: Glutamine synthetase; GOGAT: Glutamate synthase; HY5: Elongated hypocotyl 5; Inv: Invertase; MEP: Methylerythritol 4-phosphate; NR: Nitrate reductase; NiR: Nitrite reductase; NRT: Nitrate transporter; NUE: Nitrogene use efficiency; OA: Organic acid; OAA: Oxaloacetic acid; 2-OG: 2-oxoglutarate; PEPc: Phosphoenolpyruvate carboxylase; PGPR: Plant growth-promoting rhizobacteria; PK: Phosphofructokinase; pPGI: Plastidic phosphoglucose isomerase; Pyr: Pyruvate; RuBP: Ribulose 1,5-bisphosphate; Suc: Sucrose; SuSy: Sucrose synthase, TCA: Tricarboxylic acid, TF: Transcription factor.
